# The Book World of Medicine and Science

**Published:** 1904-11-12

**Authors:** 


### The Book World of Medicine and Science.

Nervous Affections of the Heart. By G. A. Gibson, M.D. (Edinburgh and London: Young J. Pentland. 1904. Pp. 99. 35 illustrations.)

Dr. Gibson's Morison lectures, delivered to the Royal College of Physicians of Edinburgh in 1902 and 1903, are well known by their previous publication in the *Edinburgh Medical Journal*, but they will be welcome to many in their present form. In his description of the sensory neuroses of the heart the author enters very fully into the question of cardiac pain and illustrates by well selected cases how variable both in its distribution and associations may be the symptom-group known as angina pectoris. In this connection there is reason to think that the stereotyped clinical picture of this condition found in the text-books requires some modification. We entirely agree with the author as to the misleading nature of the division into true and false angina as adopted by many writers. The lecture devoted to treatment is of special importance and the great clinical experience of the writer makes it an authoritative statement of our present knowledge on the subject. The section dealing with the motor disturbances of the heart is copiously illustrated by tracings in various morbid conditions. Of special interest are some tracings by Dr. Oliphant Nicholson from a case of pulsus paradoxus in which the arterial and respiratory movements are represented simultaneously in a way which brings out very clearly the alteration in the radial pulse during inspiration in this condition. The book is distinguished throughout by the freedom of style, the terseness and clearness of expression, and the evidence of wide reading which mark the successful teacher as well as the expert clinician.

The Diseases of Women: A Handbook for Students and Practitioners. By J. Bland-Sutton and Arthur E. Giles. Fourth Edition. 520 pp. 127 Illustrations. (London: Rebman, Limited. 1904.)

This small volume is so well known that the advent of a new edition calls for only a brief notice. The general character of the book has undergone little change, and by dint of some compression much recent work has been incorporated without any material increase in size. It will prove of value chiefly to students at the commencement of their work in the gynæcological wards, and to nurses who desire to know something of the scientific aspect of their profession. The teaching is dogmatic, and the views of those who differ from the authors are brushed aside without discussion; this is notably so in the chapter on uterine fibroids, and detracts considerably from the value of the book as a guide to practice. Sufficient but not too numerous references are given to original papers, and the work of British investigators receives due recognition. The book can be confidently recommended to beginners as an excellent introduction to the study of the diseases peculiar to women; and the authors are to be congratulated upon having given the maximum of information in the minimum of space without rendering the text unreadable.

Organic Nervous Diseases. By M. Allen Starr, M.D., Ph.D. (London: Baillière, Tindall and Cox. 1904. Pp. 751. With 275 illustrations and 26 plates. Price 25s. net.)

We can strongly recommend this work as a very useful addition to the physician's reference library. In the preface the author remarks that "while the extensive literature of neurology has been carefully sifted, its facts collated, and its theories considered, the endeavour has been made to utilise personal observation and experience in the presentation of each subject," and herein lies in great measure the value of the work, for it is essentially practical in character. The surgical treatment of nervous diseases is not fully entered into, except so far as the indications for operative intervention and its general scope and results are concerned. There is one criticism that is called for, namely, that the more recent work of English clinicians and pathologists has not received that amount of consideration which it deserves. The researches of Head and Mott are scarcely even referred to, with a corresponding loss, we think, to the value of the book. The numerous illustrations help the reader very materially to appreciate the descriptions given in the text, and those which show the areas of degeneration in sections of the spinal cord and the photographs of patients suffering from various forms of paralysis are beyond praise. Dr. Starr is to be congratulated on his lucid exposition of modern neurology, and on having produced a book for which there is certain to be a large demand.

Philips' Handy-Volume Atlas of London. Fourth Edition. Revised and Enlarged. (London: George Philip and Son, Ltd. Price 5s.)

This book is a good deal more than its name implies, for it includes a directory comprising some 13,000 names of streets, squares, public buildings, hospitals, etc., with references, whereby these places can be readily found on the maps; a conveyance directory which gives particulars of the railways and railway-stations, tramways, omnibuses, steamboats, and cab fares; and a great deal besides which will be of service to anyone wishing to get anywhere in London and anxious to know the best way of accomplishing his object. There are fifty-five maps which comprise the whole of the London district, and there are eleven special maps, including an index map, and others with special features. The index is entirely new and several additional maps have been added, innovations which are distinct improvements on the form of previous editions.

Baillière, Tindall and Cox.

